# Strong Dipole-Quadrupole-Exciton Coupling Realized in a Gold Nanorod Dimer Placed on a Two-Dimensional Material

**DOI:** 10.3390/nano11061619

**Published:** 2021-06-20

**Authors:** Huajian Pang, Hongxin Huang, Lidan Zhou, Yuheng Mao, Fu Deng, Sheng Lan

**Affiliations:** 1 Guangdong Provincial Key Laboratory of Nanophotonic Functional Materials and Devices, School of Information and Optoelectronic Science and Engineering, South China Normal University, Guangzhou 510006, China; hj.pang@foxmail.com (H.P.); hongxin_huang@foxmail.com (H.H.); zhould@mail2.sysu.edu.cn (L.Z.); erik_maoyh@m.scnu.edu.cn (Y.M.); 2 Department of Physics, The Hong Kong University of Science and Technology, Kowloon, Hong Kong, China

**Keywords:** two-dimensional material, scattering, strong coupling, Rabi splitting, quadrupole

## Abstract

Simple systems in which strong coupling of different excitations can be easily realized are highly important, not only for fundamental research but also for practical applications. Here, we proposed a T-shaped gold nanorod (GNR) dimer composed of a long GNR and a short GNR perpendicular to each other and revealed that the dark quadrupole mode of the long GNR can be activated by utilizing the dipole mode excited in the short GNR. It was found that the strong coupling between the dipole and quadrupole modes can be achieved by exciting the T-shaped GNR dimer with a plane wave. Then, we demonstrated the realization of strong dipole–quadrupole–exciton coupling by placing a T-shaped GNR on a tungsten disulfide (WS_2_) monolayer, which leads to a Rabi splitting as large as ~299 meV. It was confirmed that the simulation results can be well fitted by using a Hamiltonian based on the coupled harmonic oscillator model and the coupling strengths for dipole–quadrupole, dipole–exciton and quadrupole–exciton can be extracted from the fitting results. Our findings open new horizons for realizing strong plasmon–exciton coupling in simple systems and pave the way for constructing novel plasmonic devices for practical applications.

## 1. Introduction

In recent years, systems in which light is strongly coupled with matter have attracted great interest because they not only act as platforms for investigating cavity quantum electrodynamics and quantum entanglement [1,2,3,4] but also exhibit potential applications in quantum operations [5], ultrafast optical switch [6], and low threshold lasers [7]. In a photon–exciton system, strong coupling occurs when the energy exchange rate between photons and excitons is fast enough to overcome the respective energy dissipation rates. In this case, two mixed states with the characteristics of both light and matter will be generated, which are generally referred to as polaritons. This behavior is manifested in the spectrum of the system as Rabi splitting [1,4,8,9,10]. If the system is in the weak coupling regime, no polariton state will be generated and the spontaneous decay rate is governed by the so-called Purcell effect [11].

It is well known that metallic nanoparticles support localized surface plasmon resonances (LSPRs) which provide sub-wavelength confinement of light on the surfaces of nanoparticles, leading to extremely small mode volumes. In addition, the significantly enhanced electric field and the tunable resonant wavelength offered by LSPRs render them ideal platforms for realizing strong plasmon–exciton coupling. In general, the strong coupling can be revealed in the scattering spectrum of a metallic nanoparticle as an anti-crossing behavior with a large Rabi splitting exceeds the average damping rates of plasmons and excitons. Unlike bright plasmon modes, special excitation schemes are generally required for dark plasmon modes [12,13,14,15]. For example, linearly polarized light was used to excite the anapole mode of slotted silicon nanodisks and the dipole mode of gold nanorods [16,17]. Meanwhile, strong coupling between the anapole mode or dipole mode and the dark mode was observed, exhibiting a large Rabi splitting.

Recent studies have demonstrated the advantages of monolayer transition metal dichalcogenide (TMDC) in realizing strong photon–exciton or plasmon–exciton coupling because of their direct bandgaps [18], large exciton transition dipole moment [19], and extremely large absorption (MoS_2_ and WS_2_ can reach 10% and 15%, respectively at resonance) [20,21]. By designing resonant photonic structures, the absorption efficiency of TMDC can be enhanced [22]. More importantly, the binding energy of excitons in monolayer TMDCs is very large [23], implying that excitons can survive at room temperature. Thus far, the strong coupling between a TMDC monolayer and a photonic or plasmonic resonator has been successfully demonstrated, including optical microcavities [24,25], periodic nanostructures [26,27], single plasmonic nanoparticle antennas [28,29,30,31,32,33,34], and nanoparticle-on-film systems [35,36,37,38,39,40].

For a strongly coupled system consisting of a single nanoparticle and a TMDC monolayer, the typical Rabi splitting energy ranges from 80 to 120 meV [28,29,30,31]. Recently, strong coupling between excitons and the anapole mode formed by the interference of Mie resonances, which leads to a Rabi splitting of ~190 meV, has been observed in nanodisks made of multilayer WS_2_ [41]. In order to explore the rich physics of polaritons, however, it is highly desirable to further enhance the coupling strength between plasmons and excitons. On the one hand, it was predicted that the nonradiative dark mode can enhance the coupling strength between a nanocavity and quantum emitters [42]. On the other hand, the strong coupling of three excitations has become the focus of many studies. By additionally introducing a microcavity, plasmons, excitons and photons can be mixed to create three polaritonic states. When the system enters into the strong coupling regime, Rabi splitting larger than 300 or even 500 meV can be realized [25,43]. In addition, it was demonstrated that strong exciton–plasmon–polariton coupling with an effective energy separation exceeding 410 meV can be achieved by fabricating a WS_2_ grating on a gold (Au) film [44]. However, it remains a challenge to realize strong plasmon–exciton coupling with an enhanced coupling strength in a single plasmonic nanostructure on a TMDC monolayer, which can be simply characterized by measuring the scattering spectrum of the nanostructure. Very recently, strong anapole–plasmon–exciton coupling is realized by introducing a plasmonic antenna into a WSe_2_−anapole hybrid system, leading to an enhanced Rabi splitting of ~159 meV [45]. Unfortunately, complex nanofabrication techniques are necessary for realizing strong coupling in such a hybrid system.

In this article, we proposed the use of a hybrid system composed of a T-shaped GNR dimer placed on a WS_2_ monolayer to realize strong dipole–quadrupole–exciton coupling. We showed that the dark quadrupole mode in a long GNR can be excited by using the dipole mode of a short GNR placed nearby, forming a T-shaped GNR dimer. The dipole mode excited in the short GNR couples strongly with the quadrupole mode in the long GNR, resulting a Rabi splitting of ~255 meV. We demonstrated that strong dipole–quadrupole–exciton coupling can be achieved when the T-shaped dimer is placed on a WS_2_ monolayer, leading to a Rabi splitting as large as ~299 meV. The eigen-energies of the mixed states and the coupling strengths for dipole–quadrupole, dipole–exciton and quadrupole–exciton were extracted from the fitting of the numerical simulation results with the coupled oscillator model in which two and three oscillators are involved. The strong coupling is confirmed not only by an anti-crossing behavior revealed in the two-dimensional scattering and absorption spectra but also by the criterion of strong coupling.

## 2. Materials and Methods

In this work, a commercial software developed by Lumerical Solution Inc. (https://www.lumerical.com, accessed on 30 May 2021) was used for the numerical simulation based on three-dimensional finite-difference time-domain (FDTD) technique. The dielectric function of Au was taken from the experimental data [46], while that of WS_2_ was taken from previous literature [20]. The exciton energy in WS_2_ monolayer was chosen to be 2.016 eV. The diameter of the GNRs was chosen to be 40 nm and the thickness of the WS_2_ monolayer was set to be 1.0 nm. For simplicity, we considered mainly the systems suspended in air and discussed the effects of a silica (SiO_2_) substrate on the simulation results from the viewpoint of practical implementation. In the numerical simulations, we used non-uniform grids with the smallest size of 0.5 nm in all directions. In addition, a perfectly matched layer boundary condition was employed to absorb all outgoing waves.

## 3. Results and Discussion

**Figure 1:**
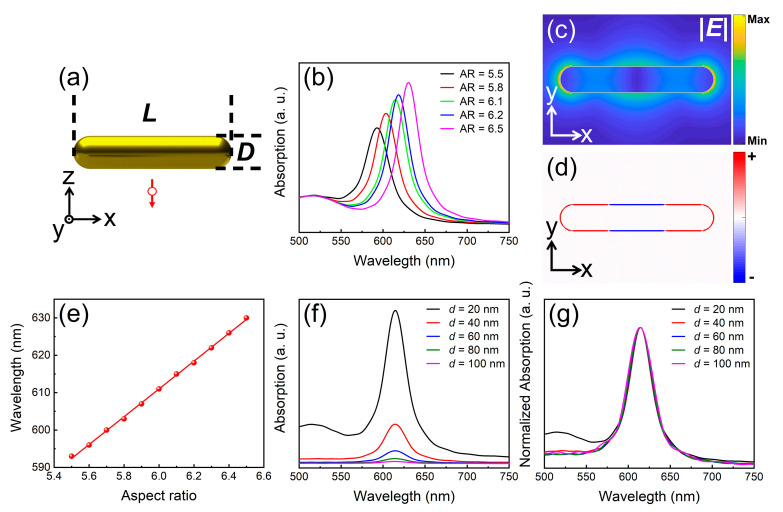
Excitation of the quadrupole mode in a GNR by using a dipole source. (**a**) Schematic showing a GNR with a length of *L* and a diameter *D* and a dipole source placed nearby. (**b**) Absorption spectra calculated for GNRs with different aspect ratios. Electric field (**c**) and surface charge (**d**) distributions calculated for a GNR with AR = 6.1 at *λ* = 615 nm. (**e**) Dependence of the resonance wavelength on the aspect ratio of the GNR. (**f**) Absorption spectra calculated for the long GNR (AR = 6.1), which is excited by a dipole source placed at different distances (*d*). (**g**) Normalized absorption spectra of the long GNR shown in (**f**).

We first investigated the excitation of the quadrupole mode supported by a GNR. Since it cannot be directly excited by using a plane wave, it is considered as a dark mode. However, it has been shown that a dipole source can be used as one of the effective ways to excite this mode [12], as schematically shown in Figure 1a. The diameter of the GNR was fixed to be *D* = 40 nm. The dipole source was placed 40 nm away from the center of the GNR and the dipole moment was made perpendicular to the long axis of the GNR.

In Figure 1b, we show the absorption spectra calculated for GNRs with aspect ratios (AR), which is defined as AR = *L*/*D*, ranging from 5.5 to 6.5. A redshift of the absorption peak as well as a narrowing of the linewidth are observed when the AR of the GNR is increased, as shown in Figure 1b. In order to verify that the absorption of the GNR arises from the excitation of the quadrupole mode, we calculated the electric field and surface charge distributions of a GNR with AR = 6.1 at the absorption peak (~615 nm), as shown in Figure 1c,d, respectively. Actually, the GNR acts as an F–P cavity which supports high-order plasmon modes with electric field distributions similar to standing waves along the long axis of the GNR [47]. The N-order plasmon mode is identified as N nodes in the electric field distribution. In Figure 1c, one can find two nodes in the electric field distribution, implying the excitation of the quadrupole mode. This assignment is further confirmed by the surface charge distribution shown in Figure 1d, which exhibits two electric dipole moments oscillating oppositely. In this case, the electric dipole of the GNR disappears completely. In Figure 1e, we present the dependence of the resonant wavelength of the quadrupole mode on the AR of the GNR, which shows a linear relationship. It means that one can readily tune the resonant wavelength of the quadrupole mode by simply varying the aspect ratio of the GNR. Then, we investigated the effects of the distance between the dipole source and the GNR surface (*d*) on the excitation of the quadrupole mode, as shown in Figure 1f,g. It was noticed that the absorption of the GNR decreases rapidly with increasing the distance. When we inspected the normalized absorption spectra, which are presented in Figure 1g, it was found that the resonant wavelength (i.e., the absorption peak) and the linewidth remained unchanged. Therefore, we conclude that the distance between the GNR and the dipole source only influences the excitation efficiency of the quadrupole mode of the GNR.

**Figure 2:**
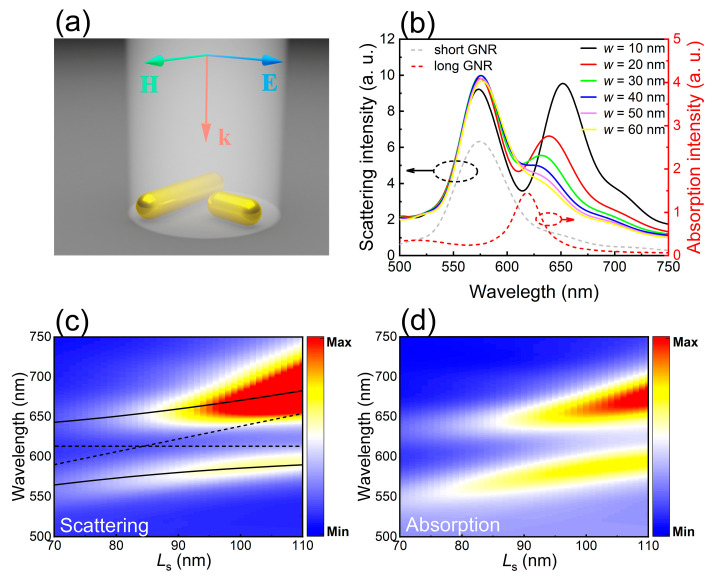
Strong coupling between the dipole and quadrupole modes excited in a T-shaped GNR dimer. (**a**) Schematic showing the T-shaped GNR dimer composed of a long and a short GNR and the wavevector and the electric/magnetic field of the plane wave used to excite the GNR dimer. (**b**) Scattering spectra calculated for GNR dimers with different gap widths (*w*). The scattering spectrum of the isolated short GNR (gray dashed curve) and the absorption spectrum of the isolated long GNR (red dashed curve) are also provided. (**c**) Two-dimensional scattering spectra calculated for GNR dimers composed of short GNRs with different lengths. The fitting results based on Equation (1) are also provided. (**d**) Two-dimensional absorption spectra calculated for GNR dimers composed of short GNRs with different lengths.

As demonstrated above, the quadrupole mode of a GNR can be excited by a dipole source placed nearby. We replaced the dipole source with another GNR with a much shorter length, forming a T-shaped GNR dimer, as shown in Figure 2a. The short GNR supports only electric dipole mode which can be easily activated by using a plane wave incident from the top. In Figure 2b, we present the scattering spectra calculated for GNR dimers with different gap widths (*w*). In this case, the lengths of the long and short GNRs are chosen to be *L*_l_ = 248 nm and *L*_s_ = 84 nm, respectively. The scattering spectrum of the isolated short GNR and the absorption spectrum of the isolated long GNR are also shown for comparison. It is noticed that the scattering spectrum of the GNR dimer, which is dominated by a single peak for a large gap width (*w* = 60 nm), evolves gradually into two peaks with identical intensities at a small gap width (*w* = 10 nm). This behavior indicates that the dipole mode of the short GNR, which is activated by the incident plane wave, excites successfully the dark quadrupole mode of the long GNR. In addition, these two modes coupled with each other, resulting in two mixed plasmon modes. The coupling strength between the two modes, which is manifested in the energy separation between the two scattering peaks, increases with decreasing gap width. The intensities of the two scattering peaks become equal when the gap width is reduced to be *w* = 10 nm, as shown in Figure 2b. As the GNR dimer is excited by using a plane wave polarized along the long axis of the short GNR, the transverse localized plasmon resonance of the long GNR, which is a dipole mode, will also be activated. It appears as a small protrusion at ~500 nm in the scattering spectrum of the GNR dimer. In Figure 2c, we show the two-dimensional scattering spectra calculated for GNR dimers composed of short GNRs with different lengths. The gap width is fixed at *w* = 10 nm. An anti-crossing behavior is clearly observed, verifying the coupling between the dipole mode of the short GNR and the quadrupole mode of the long GNR. The Hamiltonian based coupled harmonic oscillator model can be used to fit the anti-crossing behavior. It can be expressed as [48]:

(1) $$\hat{H}=\hbar \left(\begin{array}{cc} E_{D}-i\frac{\gamma_{D}}{2} & g_{D-Q}\\ g_{D-Q} & E_{Q}-i\frac{\gamma_{Q}}{2} \end{array}\right)$$

Here, $E_{D}$ and $E_{Q}$ represent the energies of uncoupled dipole and quadrupole resonances, $\gamma_{D}$ and $\gamma_{Q}$ denote the dissipation rates of the uncoupled dipole and quadrupole modes, respectively, and $g_{D-Q}$ stands for the dipole–quadrupole coupling strength. The Hopfield coefficient in each mixed state, which represents the contributions of the dipole and quadrupole modes in the mixed state, can be extracted from the Hamiltonian of the coupled system [49].

The two branches formed by dipole–quadrupole coupling can be fitted by using Equation (1), as shown in Figure 2c. The solid curves represent the high-energy ($E_{H}$) and low-energy ($E_{L}$) branches of the mixed states while the dotted lines represent the uncoupled dipole ($E_{D}$) and quadrupole ($E_{Q}$) modes. The Rabi splitting is derived to be Ω ~ 255 meV when $E_{D}=E_{Q}$. The linewidths of the $E_{D}$ and $E_{Q}$ modes used in the fitting are $\gamma_{D}$ ≈ 150 meV and $\gamma_{Q}$ ≈ 110 meV, similar to the values reported previously [47,50]. It is found that the criterion for strong coupling is satisfied in this case, i.e., $\Omega >(\gamma_{D}+\gamma_{Q})/2$ [51], implying that the coupling between the dipole and quadrupole modes enters into the strong coupling regime. As shown in Figure 2d, the anti-crossing behavior revealed in the scattering spectra is also observed in the two-dimensional absorption spectra of the GNR dimers, further confirming the strong coupling of the dipole and quadrupole modes.

**Figure 3:**
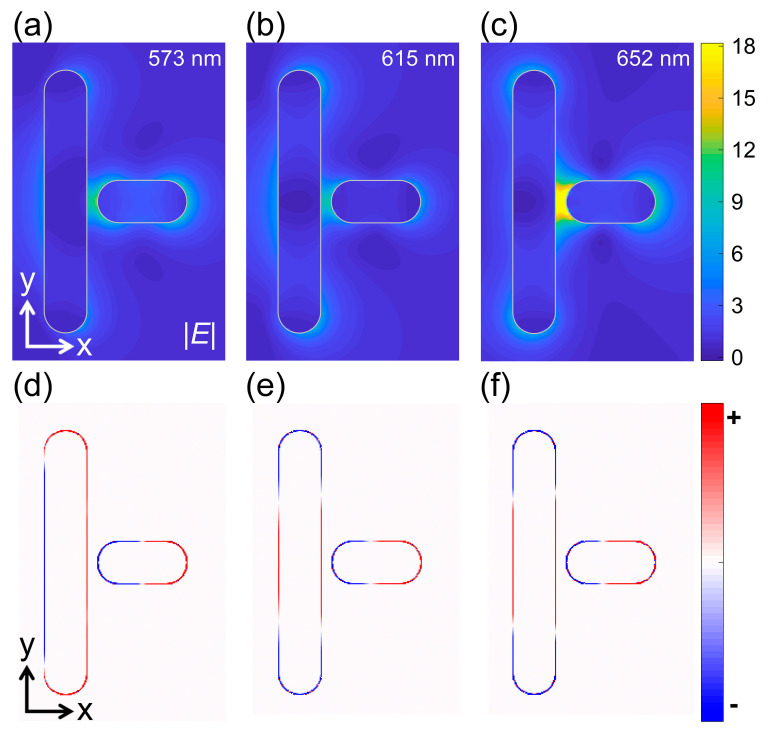
Electric field (upper panel) and surface charge (lower panel) distributions calculated for the T-shaped GNR with *L*_l_ = 248 nm, *L*_s_ = 84 nm and *w* = 10 nm at different wavelengths. (**a**,**d**) *λ* = 573 nm, (**b**,**e**) *λ* = 615 nm, (**c**,**f**) *λ* = 652 nm.

In order to gain a deep insight into the strong coupling behavior, we calculated the electric field and surface charge distributions of the GNR dimer at the scattering peaks (*λ* = 573 and 652 nm) and the scattering valley (*λ* = 615 nm), as shown in Figure 3. It was found that the electric field is localized in the gap region and the strongest one was observed at the low-energy peak (*λ* = 652 nm). The excitation of the dipole mode is also evidenced in the electric field distribution of the short GNR. The dipole mode excited in the short GNR is more clearly reflected in the surface charge distribution, as shown in Figure 3d–f. At the high-energy peak (*λ* = 573 nm), one can see the interaction between the two dipoles excited in the short and long GNRs. In contrast, a dipole–quadrupole interaction is identified at the low-energy peak (*λ* = 652 nm). It is noticed that charges with opposite signs are excited on the two sides of the gap region, implying the establishment of a strong electric field (see Figure 3c), which is crucial for realizing strong plasmon–exciton coupling described in the following.

**Figure 4:**
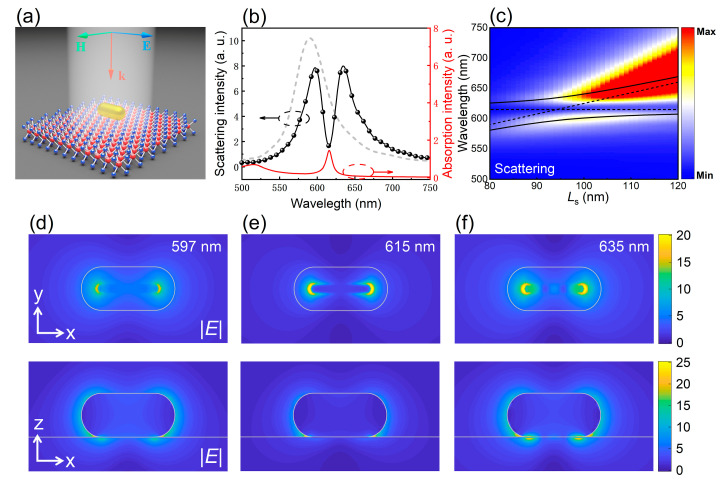
Strong coupling between the dipole mode in a single GNR and the excitons in a monolayer WS_2_. (**a**) Schematic showing the excitation of a single GNR placed on a monolayer WS_2_ by using plane wave. (**b**) Scattering spectrum (black solid curve) calculated for a GNR resonant with the exciton resonance of the monolayer WS_2_. Additionally, shown are the scattering spectrum of the GNR in the absence of the monolayer WS_2_ (dashed curve) and the absorption spectrum of the monolayer WS_2_ (red solid curve). (**c**) Two-dimensional scattering spectra calculated for GNRs with different lengths. The fitting results based on Equation (2) are also provided. (**d**–**f**) Electric field distributions in the XY plane (upper panel) and XZ plane (lower panel) calculated for the GNR with *L*_s_ = 94 nm at different wavelengths of *λ* = 597, 615 and 635 nm, respectively.

Then, we examined the coupling between the dipole mode of a GNR and the excitons in a WS_2_ monolayer. In this case, the GNR was placed on top of the monolayer WS_2_ and a plane wave whose polarization was along the axis of the GNR was incident normally on the GNR, as schematically illustrated in Figure 4a. For a GNR with *L*_s_ = 94 nm, a strong coupling between the dipole mode excited in the GNR and the excitons in the WS_2_ monolayer was achieved. As a result, the single peak in the scattering spectrum of the GNR was split into two peaks, corresponding to the two mixed states, as shown in Figure 4b. The red solid curve represents the absorption spectrum of the excitons in the WS_2_ monolayer used in the numerical simulation. It possesses a peak wavelength at ~615 nm and a linewidth of ~33 meV, consistent with the experimental data reported previously [20,30,33]. The scattering spectrum of the GNR in the absence of the WS_2_ monolayer, which is represented by the gray dotted curve, is also provided for comparison. If we plot the two-dimensional scattering spectra calculated for GNRs with different lengths, an anti-crossing behavior is clearly observed, as shown in Figure 4c. Similarly, the $E_{L}$ and $E_{H}$ branches of the plexcitons can be well fitted by using the Hamiltonian based the coupled harmonic oscillator model, which is written as:

(2) $$\hat{H}=\hbar \left(\begin{array}{cc} E_{D}-i\frac{\gamma_{D}}{2} & g_{D-\mathrm{ex}}\\ g_{D-\mathrm{ex}} & E_{\mathrm{ex}}-i\frac{\gamma_{\mathrm{ex}}}{2} \end{array}\right)$$

Here, $E_{D}$ and $E_{\mathrm{ex}}$ represent the energies of uncoupled dipole and exciton resonances, $\gamma_{D}$ and $\gamma_{\mathrm{ex}}$ denote the dissipation rates of the uncoupled dipole mode and exciton, respectively, and $g_{D-\mathrm{ex}}$ stands for the dipole–exciton coupling strengths.

The two dashed lines interacting with each other represent the energies of the dipole mode and the exciton resonance in the absence of coupling. The Rabi splitting energy extracted from the fitting is Ω ~ 123 meV, which is basically consistent with the results of previous works [28]. This value is larger than the average damping rates of the dipole mode and the exciton resonance, implying that the plasmon–exciton coupling in this system enters into the strong coupling regime. Figure 4d–f shows the electric field distributions in the XY and XZ planes at the two scattering peaks (*λ* = 597 and 635 nm) and the scattering valley (*λ* = 615 nm). It can be seen that the electric field is mainly localized on the contact surface with larger enhancements at the two ends of the GNR, indicating a strong interaction between the plasmons and the excitons.

**Figure 5:**
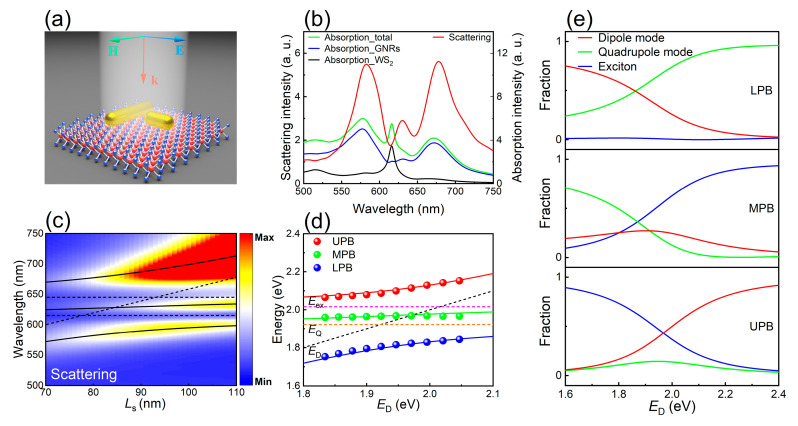
Strong dipole–quadrupole–exciton coupling realized in a T-shaped GNR dimer placed on a monolayer WS_2_. (**a**) Schematic showing a T-shaped GNR dimer placed on a monolayer WS_2_ and excited by a plane wave. (**b**) Scattering spectrum calculated for the GNR dimer placed on a WS_2_ monolayer with *L*_l_ = 248 nm, *L*_s_ = 81 nm and *w* = 10 nm. The absorption spectra of the long and short GNRs only and the absorption spectrum of the monolayer WS_2_ are also provided. (**c**) Two-dimensional scattering spectra calculated for GNR dimers composed of short GNRs with different lengths. (**d**) The red, green and blue circles represent the upper, middle and lower polariton branch (UPB, MPB and LPB) resonance energies extracted from the scattering spectrum of T-shaped GNR dimer placed on a WS_2_ monolayer hybrid structure. The red, green and blue solid lines represent the resonance energies of UPB, MPB and LPB fitted by Hamiltonian analysis Equation (3). The black, orange and pink dashed lines represent dipole, quadrupole and exciton resonances, respectively. (**e**) Hopfield coefficients for dipole, quadrupole and exciton modes contributions to UPB, MPB and LPB as a function of dipole resonance.

After discussing the dipole–quadrupole coupling and dipole–exciton coupling, we studied the dipole–quadrupole–exciton coupling in a T-shaped GNR dimer placed on a WS_2_ monolayer, as schematically illustrated in Figure 5a. In this case, the length of the short GNR was chosen to be *L*_s_ = 81 nm and the gap width was set to be *w* = 10 nm. As shown in Figure 5b, one can identify three peaks in the scattering spectrum of the GNR dimer placed on a WS_2_ monolayer. They correspond to the three mixed states arising from the strong coupling among the dipole, quadrupole and exciton modes. The intensities of the two scattering peaks are equal and they are much stronger than that of the middle one. The scattering valleys at *λ* = 615 nm and *λ* = 645 nm correspond to the exciton resonance and the quadrupole mode, respectively. The resonant wavelength of the quadrupole mode is red shifted owing to the existence of the WS_2_ monolayer which possesses a large refractive index. With increasing *L*_s_, the dipole mode is red shifted, passing through the exciton and quadrupole modes successively. One can see two anti-crossings in the two-dimensional scattering spectra plotted for GNR dimers with different *L*_s_, as shown in Figure 5c. Based on the coupled harmonic oscillator model, the Hamiltonian used to fit the three mixed states of the hybrid system can be expressed as follows [25,30,43]:

(3) $$\hat{H}=\hbar \left(\begin{array}{ccc} E_{D}-i\frac{\gamma_{D}}{2} & g_{D-Q} & g_{D-\mathrm{ex}}\\ g_{D-Q} & E_{Q}-i\frac{\gamma_{Q}}{2} & g_{Q-\mathrm{ex}}\\ g_{D-\mathrm{ex}} & g_{Q-\mathrm{ex}} & E_{\mathrm{ex}}-i\frac{\gamma_{\mathrm{ex}}}{2} \end{array}\right)$$

Here, $E_{D}$, $E_{Q}$ and $E_{\mathrm{ex}}$ represent the energies of uncoupled dipole, quadrupole and exciton resonances, $\gamma_{D}$, $\gamma_{Q}$ and $\gamma_{\mathrm{ex}}$ denote the dissipation rates of these modes, $g_{D-Q}$, $g_{D-\mathrm{ex}}$ and $g_{Q-\mathrm{ex}}$ stand for the dipole–quadrupole, dipole–exciton and quadrupole–exciton coupling strengths, respectively.

In Figure 5c, the three mixed states, which are denoted as $E_{L}$, $E_{M}$ and $E_{H}$ bands, are represented by the solid curves in the two-dimensional scattering spectra. The dotted lines represent the energies of the three uncoupled modes. It is noticed that the energies of the exciton mode ($E_{\mathrm{ex}}$ = 2.016 eV) and the quadrupole mode ($E_{Q}$ = 1.922 eV) remain nearly unchanged with increasing *L*_s_. In Figure 5d, we show the simulation results and theoretical fittings for the dipole–quadrupole–exciton coupling. Furthermore, we present the Hopfield coefficients for dipole, quadrupole, exciton contributions to the three mixed state branches of the hybrid system in Figure 5e. For the coupling of three modes, the energy splitting depends not only on the coupling strengths of dipole–quadrupole, dipole–exciton and exciton–quadrupole but also on the energies and linewidths of these modes. Therefore, the analytical expressions for the eigenenergies of the polariton modes are not available. Therefore, neither is the criterion for strong coupling. We can extract the coupling strengths between two modes to be $g_{D-Q}$ ≈ 124 meV, $g_{D-\mathrm{ex}}$ ≈ 64 meV, and $g_{Q-\mathrm{ex}}$ ≈ 32 meV based on fitting. In addition, the minimum energy splitting between the $E_{L}$ and $E_{H}$ bands is derived to be $\Omega_{H-L}$ ≈ 299 meV. This value is much larger than that observed in the coupling between a single GNR and a WS_2_ monolayer (see Figure 4), implying that the introduction of the dark quadrupole mode can really boost the plasmon–exciton coupling. Apparently, a simplified strong coupling criterion is fulfilled in this case because $\Omega_{H-L}>(\gamma_{D}+\gamma_{Q}+\gamma_{\mathrm{ex}})/2$ ≈ 146.5 meV.

**Figure 6:**
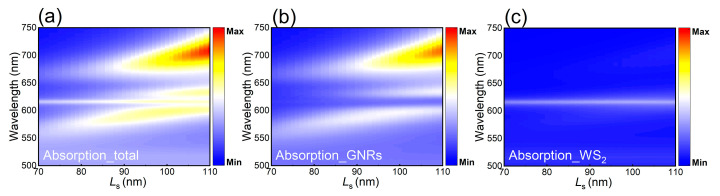
(**a**) Two-dimensional absorption spectrum calculated for a T-shaped GNR dimer with variant length of the short GNR placed on a WS_2_ monolayer. (**b**) Two-dimensional absorption spectrum calculated for a T-shaped GNR dimer with variant length of the short GNR. (**c**) Two-dimensional absorption spectrum obtained by subtracting the spectrum shown in (**b**) from that shown in (**a**).

In order to confirm the strong coupling among dipole, quadrupole and exciton realized in a T-shaped GNR dimer placed on a WS_2_ monolayer, we need to examine not only the two-dimensional scattering spectrum but also the absorption spectrum of the T-shaped GNR dimer, as shown in Figure 6a. Apart from the three branches of hybrid states, one can see a narrow absorption band at ~615 nm. However, this absorption band disappears in the two-dimensional absorption spectrum of the corresponding T-shaped GNR dimer in the absence of the WS_2_ monolayer, which is shown in Figure 6b. This phenomenon indicates that the narrow absorption band may originate from the absorption of the excitons in the WS_2_ monolayer. This assignment is verified by inspecting the two-dimensional absorption spectrum obtained by subtracting the spectrum shown in Figure 6b from that shown in Figure 6a, as shown in Figure 6c.

**Figure 7:**
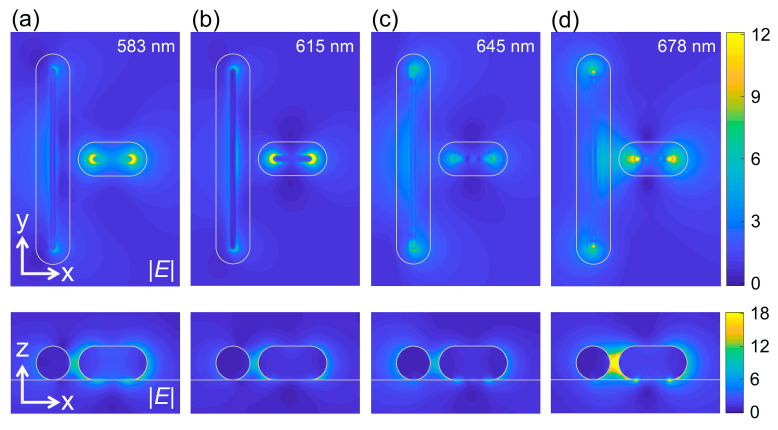
Electric field distributions in the XY (upper panel) and XZ planes (lower panel) calculated for the T-shaped GNR dimer placed on a WS_2_ monolayer with *L*_l_ = 248 nm, *L*_s_ = 81 nm and *w* = 10 nm at different wavelengths. (**a**) *λ* = 583 nm, (**b**) *λ* = 615 nm, (**c**) *λ* = 645 nm and (**d**) *λ* = 678 nm.

Next, we examined the near-fields of the three mixed states and the electric field enhancements in all polaritons modes, as shown in Figure 7. Owing to the existence of the WS_2_ monolayer, the localization of electric field occurs on the contact surface. Therefore, we can identify the dipole mode excited in the short GNR and the quadrupole excited in the long GNR. It indicates that the dark quadrupole mode is indeed excited in this system, leading to the strong coupling among the dipole, quadrupole and exciton modes. If we inspected the electric field distributions at the scattering valley of *λ* = 615 nm, we found that the electric field is mainly localized on the contact surface between the short GNR and the WS_2_ monolayer, quite similar to the coupling between a single GNR and a WS_2_ monolayer (see Figure 4). In contrast, the electric field enhancement on the contact surface between the long GNR and the WS_2_ monolayer become comparable at the scattering peaks of *λ* = 583 and 678 nm. In these cases, the electric field is mainly confined in the gap region between the two GNRs and the electric field enhancement between the long GNR and the monolayer WS_2_ is small. It means that the mode coupling in this system is dominated by dipole–exciton and dipole–quadrupole coupling, in good agreement with the coupling strengths derived from the coupled oscillator model (i.e., Equation (3)).

**Figure 8:**
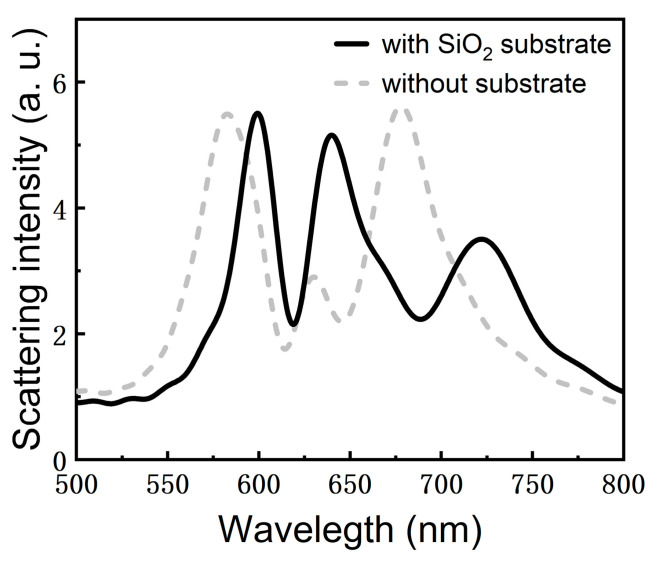
Scattering spectrum calculated for a GNR dimer placed on a SiO_2_ substrate with an embedded WS_2_ monolayer (black solid curve). The scattering spectrum of the hybrid structure in the absence of the substrate is also provided for comparison (dashed curve).

To verify the feasibility of the proposed system on practical experiments, we calculated the scattering spectrum of the GNR dimer placed on a SiO_2_ substrate, as shown in Figure 8. In this case, the SiO_2_ substrate was modeled as a semi-infinite layer with a refractive index of 1.5. In Figure 8, one can identify three scattering peaks in the scattering spectrum, implying that strong dipole–quadrupole–exciton coupling can also be realized in the presence of a SiO_2_ substrate. In addition, it is remarkable that the energy splitting between the low- and high-energy peaks appears to be larger in the presence of the SiO_2_. In indicates that strong dipole–quadrupole–exciton coupling achieved in a T-shaped GNR coupled with a WS_2_ monolayer proposed in this work can be implemented in experiments.

## 4. Conclusions

In summary, we proposed a novel strategy to enhance the plasmon–exciton coupling between the plasmons excited in GNRs and the excitons in two-dimensional materials. A T-shaped GNR dimer, which is composed of a long GNR and a short GNR arranged perpendicularly, was employed to achieve this goal through the excitation of the dark quadrupole mode in the long GNR. Three mixed states, which originate from the dipole–quadruple-exciton coupling, are revealed in both the scattering and the absorption spectra of the GNR dimer placed on a WS2 monolayer. Rabi splitting as large as 299 meV is observed, implying the strong coupling among the dipole, quadrupole and exciton modes. Our findings open new horizons for enhancing light-matter interaction and pave the wave for constructing novel nanoscale devices for practical applications.

## Data Availability

The data presented in this study are available on request from the corresponding author.
